# Oxidative Stress and Antioxidant System in Periodontitis

**DOI:** 10.3389/fphys.2017.00910

**Published:** 2017-11-13

**Authors:** Yue Wang, Oleh Andrukhov, Xiaohui Rausch-Fan

**Affiliations:** ^1^Department of Periodontology and Competence Center for Periodontal Research, University Clinic of Dentistry, Medical University of Vienna, Vienna, Austria; ^2^Department of Periodontology, Beijing Stomatological Hospital, Capital Medical University, Beijing, China

**Keywords:** oxidative stress, reactive oxygen species, antioxidants, periodontitis, neutrophils

## Abstract

Periodontitis is a common inflammatory disease, which is initiated by bacterial infection and subsequently progressed by aberrant host response. It can result in the destruction of teeth supporting tissues and have an influence on systemic health. When periodontitis occurs, reactive oxygen species, which are overproduced mostly by hyperactive neutrophils, could not be balanced by antioxidant defense system and cause tissues damage. This is characterized by increased metabolites of lipid peroxidation, DNA damage and protein damage. Local and systemic activities of antioxidants can also be influenced by periodontitis. Total antioxidant capacity, total oxidant status and oxidative stress index have been used to evaluate the oxidative stress associated with periodontitis. Studies have confirmed that inflammatory response in periodontitis is associated with an increased local and systemic oxidative stress and compromised antioxidant capacity. Our review focuses on increased oxidative stress in periodontal disease, specifically, on the relationship between the local and systemic biomarkers of oxidative stress and periodontitis and their association with the pathogenesis of periodontitis. Also, the relationship between periodontitis and systemic inflammation, and the effects of periodontal therapy on oxidative stress parameters will be discussed.

## Introduction

Periodontitis is a prevalent inflammatory disease, influencing at least 10% of people worldwide (Richards, 2014). It can result in the destruction of teeth supporting tissue and ends up with a loss of teeth. In addition, periodontitis has been suggested to have moderate association with several systemic diseases, e.g., cardiovascular disease, diabetes, and adverse pregnancy outcomes (Nazir, 2017). Current concept suggests that this inflammatory disease is initiated by bacterial infection and subsequently progressed by aberrant host response, which mainly contributes to periodontal tissue destruction (Bartold and Van Dyke, 2013).

In recent years, reactive oxygen species (ROS) have gained more and more attention, because of their central role to the progression of many inflammatory diseases (Mittal et al., 2014). ROS are described as oxygen free radicals and other non-radical oxygen derivatives involved in oxygen radical production (Lushchak, 2014). They are involved in normal cellular metabolism and continuously generated by the cells in most tissues. Another category of substances called antioxidants exist in the cells and can effectively delay or inhibit ROS-induced oxidation (Sies, 1997). Under physiological conditions, ROS are effectively neutralized by antioxidants, which prevent ROS-mediated tissue damage. When inflammation happens, ROS production is drastically increased mainly due to cells of innate immune system, e.g., neutrophils and macrophages during the process of phagocytosis via the metabolic pathway of the “respiratory burst” (Mittal et al., 2014). Subsequently, high levels or activities of ROS cannot be balanced by the antioxidant defense system, which leads to the oxidative stress and tissue damage (Sies, 1997). ROS can directly cause tissue damage, involving lipid peroxidation, DNA damage, protein damage, and oxidation of important enzymes; meanwhile, they can function as signaling molecules or mediators of inflammation (Chapple and Matthews, 2007).

Over the past few years, numerous clinical and basic experimental studies have shown a strong association between oxidative stress and periodontitis. Getting a better understanding of this association can give us a deeper insight into the pathogenesis of periodontitis, relationship between periodontitis and systemic inflammation, and therapeutic strategies. Therefore, the aim of this review is to summarize the current findings of the association between local and systemic oxidative stress and periodontitis.

## Overproduction of ROS associated with periodontitis

**Figure 1:**
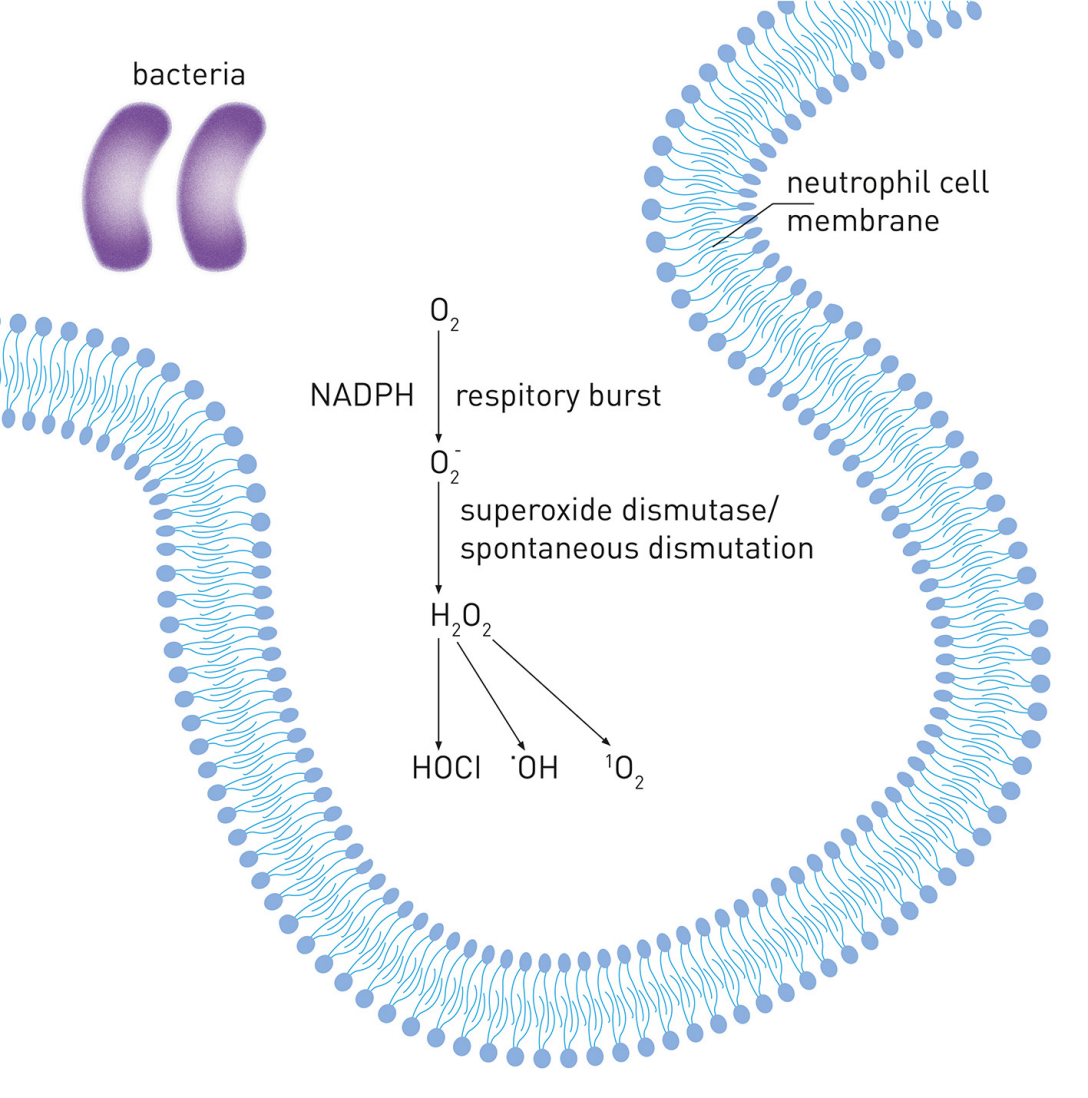
Reactive oxygen species production in periodontal disease. Upon internalization of pathogens, neutrophils produce ${\text{O}}_{2}^{-}$ via the metabolic pathway called “respiratory burst” by NADPH-oxidase. ${\text{O}}_{2}^{-}$ can be converted to hydrogen peroxide (H_2_O_2_) by superoxide dismutase or spontaneous dismutation. H_2_O_2_ can be further converted to different derivatives, such as hypochlorous acid (HOCl), hydroxyl radical (^•^OH) and singlet oxygen (^1^O_2_).

Neutrophils are the most abundant blood white cells and belong to first defense line against bacterial infection. After initiation of the host response by pathogenic biofilm, neutrophils become the most common inflammatory cells gathering in periodontal tissue and gingival sulcus and they are believed to be the predominant source of ROS in periodontitis (Miyasaki, 1991). Following the stimulation by pathogens, neutrophils produce ${\text{O}}_{2}^{-}$ via the metabolic pathway called “respiratory burst” catalyzed by NADPH oxidase during phagocytosis (Chapple and Matthews, 2007). ${\text{O}}_{2}^{-}$ can be released into phagosomal and extracellular environment and then converted to different radical and non-radical derivatives, such as hydrogen peroxide (H_2_O_2_), hypochlorous acid (HOCl), hydroxyl radical (OH•) and singlet oxygen (^1^O_2_). Figure 1 shows the mechanisms of increased ROS production in periodontal disease.

Numerous studies focused on the peripheral blood neutrophils of periodontitis patients and showed that their activity of producing ROS is higher compared to neutrophils from healthy individuals (Gustafsson and Asman, 1996; Fredriksson et al., 1998, 2003; Gustafsson et al., 2006; Matthews et al., 2007a,b; Wright et al., 2008; Aboodi et al., 2011; White et al., 2014; Ling et al., 2016). Consistent results have shown that peripheral blood neutrophils of people with chronic periodontitis (CP) or aggressive periodontitis (AgP) generate significantly more ROS upon simulation with purified immunoglobulin opsonized *Staphylococcus aureus* compared with peripheral blood neutrophils of healthy controls suggesting that people with periodontitis have a hyper-reactive phenotype of neutrophils and these neutrophils can be stimulated by the Fc-gamma receptor (FcγR) pathway (Gustafsson and Asman, 1996; Fredriksson et al., 1998; Gustafsson et al., 2006; Matthews et al., 2007a). One study by Fredriksson et al confirmed that increased ROS production by neutrophils of periodontitis patients occurs via the stimulation of FcγR pathway and not via complement receptor CR3 or intracellular protein kinase C enzyme (Fredriksson et al., 2003). Hyper-reactivity of both periodontitis patients and control neutrophils was also shown upon the stimulation of unopsonized periodontal pathogen *Fusobacterium nucleatum* (Matthews et al., 2007a). It has been shown that even without any stimulation neutrophils of periodontitis patients release more extracellular ROS than neutrophils of healthy controls (Matthews et al., 2007a; Ling et al., 2016). One longitudinal study showed that periodontal therapy could reduce FcγR-stimulated (with/without priming with *Porphyromonas gingivalis* and *F. nucleatum*) ROS production, but had no effect on unstimulated extracellular ROS (Matthews et al., 2007b). The same study observed that unstimulated ROS production was higher in periodontitis patients than in healthy controls therefore it was concluded that both constitutive and reactive mechanisms contribute to the hyperreactivity of neutrophils in periodontitis (Matthews et al., 2007b). A recent study demonstrated that peripheral blood neutrophils of CP patients produced more extracellular superoxide with or without stimulation of unopsonized *F. nucleatum, P. gingivalis* and phorbol myristate acetate and this superoxide overproduction was reduced upon non-surgical therapy, indicating that the hyperactivity of neutrophil is related to both reactive and constitutional mechanisms (Ling et al., 2016). Additionally, the level of superoxide released by unstimulated pre-therapy neutrophils significantly positively correlated with the level of C-reactive protein in plasma (Ling et al., 2016). This correlation might be partially explained by the fact that CRP increases toll-like receptor(s) induced superoxide released by neutrophils thus increasing oxidative stress (Ling et al., 2014). There are also studies suggesting an association between NADPH oxidase and FcγR polymorphism and periodontitis (Nibali et al., 2006; Dimou et al., 2010). These studies support the idea that an increased ROS generation in periodontitis could be not only due to stimulation by pathogens but also is genetically predisposed (Giannopoulou et al., 2008).

*In vitro* studies show that not only neutrophils but also other phagocytes and cells of periodontal tissues, e.g., monocytes, gingival fibroblasts and periodontal ligament cells exhibit enhanced ROS production upon stimulation by periodontal pathogens and/or their components (Bullon et al., 2011; Chang et al., 2013; Golz et al., 2014). However, their contribution into oxidative stress in periodontitis still remains to be elucidated by future studies.

## Metabolic products of ROS in periodontitis

ROS are very active and their life time is extremely short. They can cause direct damage to the tissues resulting in a variety of metabolites of lipid peroxidation, DNA damage, and protein damage, which are usually used to evaluate the destruction of tissue by ROS (Chapple and Matthews, 2007).

### Lipid peroxidation

Lipid peroxidation products are the most investigated derivatives of ROS in periodontitis. Lipid peroxidation by free radicals results in the changes of structural integrity and function of cell membranes. Several products of lipid peroxidation such as malondialdehyde (MDA), 4-hydroxyl-2-115 nonenal (HNE), and isoprostane have been used to evaluate both local and systemic oxidative damages associated with periodontitis. Table 1 summarizes the studies on the relationship between lipid peroxidation products and periodontitis.

**Table 1:** Studies investigating the relationship between lipid peroxidation products and periodontitis.

**Analyzed markers**	**Biological samples**	**Participants**	**Results**	**References**
TBARS	Plasma; erythrocytes; erythrocyte membranes; gingival tissues	25 CP; 25 controls	↑TBARS in CP for all types of samples (*p* < 0.001)	Panjamurthy et al., 2005
TBARS	Saliva	217 dental patients	↑TBARS correlates with ↑BI (*p* < 0.001)	Celec et al., 2005
TBARS	Saliva	204 dental patients	↑TBARS correlates with ↑BI (*p* < 0.026) and ↑age (*p* < 0.006)	Celecova et al., 2013
TBARS	Saliva	115 pregnant women; 72 non-pregnant women	↓TBARS after giving birth than being pregnant; ↑TBARS correlates with ↑ probing depth (*p* < 0.001), ↑ clinical attachment level (*p* < 0.003), ↑ bleeding on probing (*p* < 0.016), ↑ plaque index (*p* < 0.001) in non-pregnant women	Gumus et al., 2015
TBARS	Saliva; blood	55 CP; 55 controls	↑TBARS in CP for all types of samples (*p* = 0.0001); no influence of gender	Ahmadi-Motamayel et al., 2017
TBARS	Saliva	23 CP; 19 controls	↑TBARS in male CP than male controls (*p* < 0.01)	Banasova et al., 2015
TBARS	Saliva	82 pediatric dental patients	↑TBARS correlates with ↑BI in children (*p* < 0.05)	Tothova et al., 2013
MDA	GCF; saliva	13 CP; 9 controls	↑MDA of GCF (*p* < 0.005) and saliva (*p* < 0.05) in CP than controls, and decreases after therapy (*p* < 0.05)	Tsai et al., 2005
MDA	GCF; saliva; serum	65 CP; 35 controls	↑MDA of GCF in CP than controls (*p* < 0.05), and decreases after therapy (*p* < 0.05)	Wei et al., 2010
MDA	Gingival tissue; serum	49 CP (23 smokers, 23 former smokers, 20 non-smokers); 20 controls (non-smokers)	↑MDA of both types of samples in CP than controls (*p* < 0.01); ↑MDA in CP (smokers) than CP (former smokers and non-smokers) (*p* < 0.01).	Tonguc et al., 2011
MDA	GCF	25 CP; 25 General AgP; 15 controls	Concentration of MDA: General AgP > CP > controls (*p* < 0.001).	Ghallab et al., 2016
MDA	GCF; saliva; serum	36 CP; 28 controls	↑MDA of GCF and saliva in CP than controls (*p* < 0.05).	Akalin et al., 2007
MDA	Saliva	30 CP (15 smokers, 15 non-smokers); 30 controls (15 smokers, 15 non-smokers)	↑MDA in CP (smokers) than controls (non-smokers) (*p* < 0.05), and decreases after therapy (*p* < 0.05).	Guentsch et al., 2008
MDA	Saliva	30 CP; 30 controls	↑MDA in CP than controls (*p* < 0.001).	Canakci et al., 2009
MDA	Saliva	20 CP; 20 controls	↑MDA in CP than controls (*p* < 0.05)	Miricescu et al., 2014
MDA	Saliva	33 CP; 16 gingivitis; 37 controls	↑MDA in CP than controls and gingivitis, and correlates with the percentage of bleeding on probing and presence of periodontal pathogens.	Almerich-Silla et al., 2015
MDA	Saliva; serum	30 CP; 35 general AgP; 30 controls	↑MDA of GCF in CP and general AgP than controls (*p* < 0.05), and correlates with clinical parameters.	Baltacioglu et al., 2014b
MDA	Saliva; plasma	60 CP (30 with type 2 diabetics, 30 systemically healthy); 60 controls (30 with type 2 diabetics, 30 systemically healthy)	↑MDA in CP (*p* < 0.05); no difference between CP with type 2 diabetics and systemically healthy CP	Trivedi et al., 2014
MDA	Blood	37 CP (18 with hyperlipidemia, 19 systemically healthy); 37 controls (18 with hyperlipidemia, 19 systemically healthy)	↑MDA in CP (with hyperlipidemia) than CP (systemically healthy) and controls (systemically healthy).	Fentoglu et al., 2015
MDA	Saliva	32 CP (16 with acute coronary syndrome, 16 systemically healthy); 32 controls (16 with acute coronary syndrome, 19 systemically healthy)	↑MDA in CP (with acute coronary syndrome) than CP (systemically healthy) and controls; MDA correlates with clinical parameters.	Nguyen et al., 2016
MDA	Saliva	217 dental patients	↑MDA of saliva in smokers than non-smokers (*p* < 0.003)	Celec et al., 2005
MDA	GCF; saliva; serum	25 CP; 26 controls	↑MDA of saliva in CP than controls (*p* < 0.001), No change at 3-weeks after therapy (*p* < 0.05)	Onder et al., 2017
HNE	GCF; saliva; serum	47 CP (24 smokers, 23 non-smokers); 46 controls (23 smokers, 23 non-smokers)	↑HNE of GCF in CP(smokers) than controls (non-smokers)(*p* = 0.001), No change at 3-months after therapy (*p* < 0.05)	Hendek et al., 2015
HNE	Saliva; serum	25 CP; 26 controls	↑HNE of serum in CP than controls (*p* < 0.001), No change at 6-weeks after therapy (*p* < 0.05)	Onder et al., 2017
HNE	Saliva; serum	30 CP (15 with type 2 diabetics, 15 systemically healthy); 10 controls (systemically healthy)	HNE concentration: CP with type 2 diabetics > systemically healthy CP >controls (*p* < 0.05)	Pradeep et al., 2013a
Isoprostane	GCF	26 CP; 26 gingivitis; 26 controls	8-Isoprostane concentration: CP >gingivitis >controls (*p* < 0.001)	Pradeep et al., 2013c
Isoprostane	Saliva	121 adults (31 smokers, 90 non-smokers)	↑8-epi-prostaglandin F2 alpha in smokers than non-smokers (*p* < 0.0001), and correlates with plaque index.	Wolfram et al., 2006
Isoprostane	Saliva	58 CP; 234 controls	↑8-epi-prostaglandin F2 alpha in CP than controls (*p* < 0.0001)	Su et al., 2009

*TBARS, thiobarbituric acid reacting substances; MDA, malondialdehyde; HNE, 4-hydroxyl-2-nonenal; GCF, gingival crevicular fluid; CP, chronic periodontitis; AgP, aggressive periodontitis; BI, bleeding index*.

#### MDA

MDA is a well-established lipid peroxidation product to evaluated oxidative stress, and it is also the most investigated lipid peroxidation product in periodontitis (Ahmadi-Motamayel et al., 2017).

Thiobarbituric acid reacting substances (TBARS) is a conventional method to detect MDA based on the reaction with thiobarbituric acid and measured by spectrophotometric assay (Yagi, 1976). It must be noted that this method is not specific for MDA and might also detect other aldehydes, which are also reactive with thiobarbituric acid and produce compound with similar absorption wavelengths as MDA (Halliwell and Whiteman, 2004). It has been shown that periodontitis is associated with higher levels of TBARS in blood plasma and erythrocytes systemically, as well as in gingival crevicular fluid (GCF) and gingival tissue locally (Panjamurthy et al., 2005). The association between increased TBARS levels and deteriorating periodontal status has been also shown in saliva of adults (Celec et al., 2005; Celecova et al., 2013; Gumus et al., 2015; Ahmadi-Motamayel et al., 2017), especially in men (Banasova et al., 2015), and children (Tothova et al., 2013).

Liquid chromatography and mass spectroscopy are more reliable and specific methods for the detection of MDA (Akalin et al., 2007). These methods were used to study MDA levels in serum, GCF, and saliva of periodontitis patients (Tsai et al., 2005; Akalin et al., 2007; Wei et al., 2010). Significantly higher levels of MDA were found in GCF and gingival tissue of periodontitis patients compared to periodontal healthy controls (Tsai et al., 2005; Wei et al., 2010; Tonguc et al., 2011). Moreover, a study by Ghallab et al. demonstrated that levels of MDA in GCF could discriminate between general AgP, CP, and periodontally healthy controls (Ghallab et al., 2016). Salivary MDA levels in periodontitis were extensively investigated and most of the studies showed higher salivary MDA in periodontitis patients compared to periodontally healthy controls (Tsai et al., 2005; Akalin et al., 2007; Guentsch et al., 2008; Canakci et al., 2009; Wei et al., 2010; Baltacioglu et al., 2014b; Miricescu et al., 2014; Trivedi et al., 2014; Almerich-Silla et al., 2015; Onder et al., 2017). A study by Baltacioglu et al. compared the level of MDA in saliva between people with AgP, CP, and periodontally healthy controls and found that AgP and CP groups have significantly higher levels of MDA than control group, but no differences between AgP and CP groups were observed (Baltacioglu et al., 2014b). It has also been shown that the higher local levels of MDA in periodontitis patients can be diminished upon periodontal therapy (Tsai et al., 2005; Guentsch et al., 2008; Wei et al., 2010). There are also some studies investigating the level of MDA in serum of periodontitis patients; however, in contrast to the data on local MDA levels, their results are controversial (Akalin et al., 2007; Wei et al., 2010; Baltacioglu et al., 2014b; Trivedi et al., 2014; Fentoglu et al., 2015; Onder et al., 2017). Two studies, in which MDA levels were measured in GCF, saliva, and serum of CP patients, showed that periodontitis had no effect on systemic MDA levels, although local MDA levels were increased in periodontitis patients (Akalin et al., 2007; Wei et al., 2010). This finding suggests that the influence of periodontitis on systemic oxidative stress might be limited. However, a meta-analysis performed by Liu et al included 5 studies on systemic MDA in periodontitis and showed that periodontitis patients had higher level of circulating MDA than healthy controls (Liu et al., 2014). Recently, a study with rather large sample size (55 CP and 55 healthy controls) also confirmed the significant difference of MDA level in serum between CP and healthy controls (Ahmadi-Motamayel et al., 2017). Meanwhile, studies including patients with diabetes mellitus, hyperlipidemia and acute coronary syndrome indicated that periodontitis could also contribute to higher circulating level of MDA among people with these systemic diseases (Trivedi et al., 2014; Fentoglu et al., 2015; Nguyen et al., 2016). Smoking is one of the most important risk factors for periodontitis and several studies showed that systemic and local MDA levels were increased by smoking independently on the impact of periodontitis (Celec et al., 2005; Guentsch et al., 2008; Tonguc et al., 2011). All above data suggest that MDA may reflect increased local and systemic oxidative stress associated with periodontitis in combination with either systemic disease or smoking.

#### HNE

HNE is another major aldehydes end product associated with lipid peroxidation (Petersen and Doorn, 2004) but data on this biomarker in periodontitis are limited to date. A study by Hendek et al. investigated the impact of periodontitis, smoking and periodontal treatment on HNE levels in GCF, saliva, and serum, and found significant different GCF levels of HNE between smokers with periodontitis and periodontally healthy non-smokers (Hendek et al., 2015). In contrast to this study, Onder et al. showed that the levels of HNE are increased by periodontitis only in serum but not in saliva (Onder et al., 2017). Both of the above studies did not show the reduction of HNE level after periodontal treatment. A study detecting HNE modified histidine adducts showed that the level of HNE-Histidine adducts in both GCF and serum were significantly increased in periodontitis with or without diabetes mellitus (Pradeep et al., 2013a).

#### Isoprostane

Isoprostane is a product of arachidonic acid peroxidation and is often measured in urine, serum or plasma as a reliable marker of oxidative stress (Roberts and Morrow, 2002). There are few studies investigating isoprostane levels in periodontitis (Wolfram et al., 2006; Su et al., 2009; Pradeep et al., 2013c). Elevated salivary levels of 8-epi-prostaglandin F2 alpha, one of isoprostanes, were associated with periodontal disease severity and were significantly increased by smoking (Wolfram et al., 2006; Su et al., 2009). Another study by Pradeep et al. (2013c) showed that 8-isoprostane levels in GCF increased progressively from healthy controls to gingivitis and periodontitis and correlated with gingival index, probing depth, and clinical attachment level (Pradeep et al., 2013c). All the above studies indicated that specific isoprostanes could be promising oxidative stress markers for periodontitis, and further longitudinal and prospective studies with a larger population are required.

### Protein damage

ROS can cause fragmentation of polypeptides or covalent crosslinking resulting in changes of protein functional activity (Shacter, 2000). Some protein damage by ROS was investigated in periodontitis. Table 2 summarizes the studies investigating the relationship between protein damage products and periodontitis.

**Table 2:** Studies investigating the relationship between protein damage products and periodontitis.

**Analyzed markers**	**Biological samples**	**Participants**	**Results**	**References**
PC	GCF	25 CP; 25 gingivitis; 25 controls	↑PC in CP than gingivitis and controls, and correlates with clinical parameters.	Pradeep et al., 2013b
PC	Saliva	48 CP (24 with acute coronary syndrome, 24 systemically healthy); 48 controls (24 with acute coronary syndrome, 24 systemically healthy)	↑PC in CP and controls with acute coronary syndrome than systemically healthy controls (*p* < 0.05).PC correlates with probing depth, plaque index (*p* < 0.05).	Nguyen et al., 2017
PC	Saliva	58 CP; 234 controls	↑PC and ↑specific oxidation of transferrin, human IgG1 heavy chain fragment, and amylase in CP than controls (*p* < 0.0001)	Su et al., 2009
AOPP	Saliva	204 dental patients	AOPP doesn't correlates with BI, and correlates with caries.	Celecova et al., 2013
AOPP	Saliva	82 pediatric dental patients	AOPP doesn't correlates with BI, and correlates with caries.	Tothova et al., 2013
AOPP	Saliva	23 CP; 19 controls	No difference of AOPP between groups	Banasova et al., 2015

*PC, protein carbonyls; AOPP, advanced oxidation protein products; GCF, gingival crevicular fluid; CP, chronic periodontitis (CP); AgP, aggressive periodontitis; BI, bleeding index*.

#### Protein carbonyl groups

Protein carbonyl (PC) groups are relatively stable end-products of protein oxidation generated by multiple forms of ROS. It is the most widely used biomarker for oxidative protein damage with earlier production and greater stability compared with lipid peroxidation products (Frijhoff et al., 2015). The association between periodontal status and PC groups has been investigated in GCF, saliva and serum and higher levels of PC groups were associated with worse periodontal status, as well as significant correlation between the level of PC groups and clinical periodontal parameters was observed within periodontitis patients (Sculley and Langley-Evans, 2003; Baltacioglu et al., 2008; Pradeep et al., 2013b; Nguyen et al., 2017). One study even showed that some specific salivary proteins such as transferrin, human IgG1 heavy chain fragment, and amylase exhibited higher oxidation levels in periodontitis compared to healthy controls (Su et al., 2009).

#### Advanced oxidation protein products

Advanced oxidation protein products (AOPP) is also thought to be a sensitive biomarker of protein oxidation, especially related to the activation of neutrophil and the activity of myeloperoxidase (Witko-Sarsat et al., 1996). AOPP have been detected in saliva, however, no relationship was found between their levels and periodontal status among adults or children (Celecova et al., 2013; Tothova et al., 2013; Banasova et al., 2015).

### DNA damage

ROS can react with DNA and cause damage to purine and pyrimidine bases or the deoxyribose backbone (Halliwell, 2000). 8-Hydroxy-deoxyguanosine (8-OHdG) is most often used biomarker of oxidative stress-induced DNA damage, although it may not precisely reflect the whole DNA damage resulting from oxidative stress (Chapple and Matthews, 2007). Table 3 summarizes the studies on the relationship between DNA damage products and periodontitis.

**Table 3:** Studies investigating the relationship between DNA damage products and periodontitis.

**Analyzed markers**	**Biological samples**	**Participants**	**Results**	**References**
8-OHdG	saliva	30 CP; 30 controls	↑8-OHdG in CP (*p* < 0.001)	Canakci et al., 2009
8-OHdG	saliva	58 CP; 234 controls	↑8-OHdG in CP (*p* = 0.0003); 8-OHdG negatively correlates with Community Periodontal Index of Treatment Needs (*p* = 0.004)	Su et al., 2009
8-OHdG	saliva	20 CP; 20 gingivitis; 20 controls	↑8-OHdG in CP than gingivitis and controls (*p* < 0.001), correlates with age (*p* < 0.05), probing depth (*p* < 0.001) and CAL (*p* < 0.001)	Sezer et al., 2012
8-OHdG	GCF; saliva	24 CP; 24 controls	↑8-OHdG of GCF in CP (*p* < 0.001), decreases after therapy (*p* < 0.001); 8-OHdG of saliva doesn't differ between groups or after therapy.	Dede et al., 2013
8-OHdG	GCF; saliva; serum	47 CP (24 smokers, 23 non-smokers); 46 controls (23 smokers, 23 non-smokers)	↑8-OHdG of GCF in CP than controls (*p* < 0.001); ↑8-OHdG of saliva in CP than controls (non-smokers) (*p* < 0.003).	Hendek et al., 2015
8-OHdG	saliva	23 CP; 25 controls	↑8-OHdG correlates with clinical parameters (*p* < 0.001), and decreases after therapy (*p* < 0.001)	Kurgan et al., 2015
8-OHdG	saliva	58 CP; 42 AgP; 60 controls	↑8-OHdG in CP and AgP than controls (*p* < 0.05)	Zamora-Perez et al., 2015
8-OHdG	saliva; serum	25 CP; 26 controls	↑8-OHdG of saliva in CP than controls (*p* < 0.001), and decreases after therapy (*p* < 0.001).	Onder et al., 2017
8-OHdG	saliva	211 adults	↑8-OHdG correlates with periodontitis.	Shin et al., 2016
8-OHdG	GCF; saliva; plasma	45 obese individuals; 45 normal-weight individuals	↑8-OHdG of all types of samples in CP than controls (*p* < 0.05); ↑8-OHdG of plasma in obese individuals with periodontitis than normal-weight individuals (*p* < 0.05); 8-OHdG in CP and gingivitis decreases after therapy (*p* < 0.01).	Ongoz Dede et al., 2016
8-OHdG	saliva	45 CP; 47 controls	↑8-OHdG in CP than controls, correlates with clinical parameters and quantity of periodontal pathogens, and decreases after therapy.	Yang et al., 2016
8-OHdG	saliva	38 patients	↑8-OHdG in patients positive for S. anginosus, and decreases after therapy.	Sugano et al., 2003
8-OHdG	saliva	29 periodontitis; 20 controls	↑8-OHdG in CP than controls, correlates with quantity of P. gingivitis (*p* < 0.01), decreases after therapy (*p* < 0.01).	Sawamoto et al., 2005
8-OHdG	saliva	33 CP; 16 gingivitis; 37 controls	↑8-OHdG in CP than controls and gingivitis, and correlates with the percentage of bleeding on probing and presence of periodontal pathogens.	Almerich-Silla et al., 2015
8-OHdG	saliva	115 pregnant women; 72 non-pregnant women	↑8-OHdG in pregnant women than non-pregant women; ↑8-OHdG correlates with↑probing depth (*p* < 0.001), ↑CAL (*p* < 0.001) after partum.	Gumus et al., 2015
8-OHdG	blood	37 CP (18 with hyperlipidemia, 19 systemically healthy); 37 controls (18 with hyperlipidemia, 19 systemically healthy)	↑8-OHdG in CP with hyperlipidemia than systemically healthy CP and systemically healthy controls.	Fentoglu et al., 2015

*8-OHdG, 8-Hydroxy-deoxyguanosine; GCF, gingival crevicular fluid; CP, chronic periodontitis (CP); AgP, aggressive periodontitis*.

Numerous studies showed higher level of 8-OHdG in GCF and saliva of periodontitis patients compared with that of healthy controls as well as their significant association with clinical periodontal parameters (Takane et al., 2002; Canakci et al., 2009; Su et al., 2009; Sezer et al., 2012; Dede et al., 2013; Hendek et al., 2015; Kurgan et al., 2015; Zamora-Perez et al., 2015; Shin et al., 2016; Onder et al., 2017). 8-OHdG levels are significantly reduced by periodontal treatment (Takane et al., 2002; Dede et al., 2013; Hendek et al., 2015; Kurgan et al., 2015; Ongoz Dede et al., 2016; Yang et al., 2016; Onder et al., 2017). However, there is no difference in the local levels of 8-OHdG between individuals with gingivitis and periodontitis (Sezer et al., 2012), as well as between CP and AgP patients (Zamora-Perez et al., 2015). A recent study suggested that liquid chromatography tandem mass spectrometry is a more sensitive approach to evaluate the levels of 8-OHdG in saliva with reliability similar to the conventional enzyme linked immune sorbent assay (Kurgan et al., 2015). However, this method of 8-OHdG detection needs to be applied for other samples such as GCF and plasma. Several studies indicated that the level of 8-OHdG is associated with the presence and/or quantity of bacteria such as *P. gingivalis, Tannerella forsythia, Treponema denticola* and *Streptococcus anginosus* (Sugano et al., 2003; Sawamoto et al., 2005; Almerich-Silla et al., 2015; Yang et al., 2016). Recently, studies have shown that the levels of 8-OHdG in saliva is significantly elevated by pregnancy and smoking (Gumus et al., 2015; Kurgan et al., 2015). Moreover, studies investigating the serum levels of 8-OHdG showed that it could be influenced by several systemic conditions such as obesity and hyperlipidemia independently on periodontitis (Fentoglu et al., 2015; Hendek et al., 2015; Onder et al., 2017). Based on above mentioned studies, we can conclude that local levels of 8-OHdG are closely related to periodontitis with some impact of systemic conditions, whereas the systemic levels of 8-OHdG depend more on systemic conditions than on periodontal status.

## Antioxidant

Under normal physiological conditions, there is a balance between ROS and antioxidants. Oxidative stress happens only when the antioxidant defense system could not neutralize the elevated ROS production (Sies, 1997). Antioxidants can be classified as two categories based on their mode of function (Chapple and Matthews, 2007). First category comprises preventive antioxidants including enzymatic antioxidants such as superoxide dismutase (SOD), catalase (CAT), glutathione peroxidase (GPx), glutathione reductase, and DNA repair enzymes, as well as some metal ion sequestrators such as albumin. Second category comprises scavenging antioxidants or chain breaking antioxidants such as ascorbic acid (vitamin C), carotenoids (including retinol-vitamin A), uric acid, α-tocopherol (vitamin E), reduced glutathione, and polyphenols (flavenoids). Table 4 summarizes the studies investigating the relationship between antioxidants and periodontitis.

**Table 4:** Studies investigating the relationship between antioxidants and periodontitis.

**Analyzed markers**	**Biological samples**	**Participants**	**Results**	**References**
SOD; CAT	Gingival tissue	< or = 3 mm; 4–6 mm; >6 mm gingival tissues	↓SOD and ↓CAT in >6 mm than other groups	Ellis et al., 1998
SOD; GPx	Saliva	30 CP; 30 controls	↓SOD and ↓GPx in CP (*p* < 0.05); SOD and GPx negatively correlates with MDA and 8-OHdG (*p* < 0.001)	Canakci et al., 2009
SOD; CAT; glutathione reductase	Saliva	30 CP; 30 controls	↓SOD, ↓CAT, and ↓glutathione reductase in CP; SOD, CAT, and glutathione negatively correlates with clinical parameters.	Trivedi et al., 2015
SOD; CAT; GPx; glutathione reductase; vitamin C; vitamin E	Plasma; erythrocytes; erythrocyte membranes; gingival tissues	25 CP; 25 controls	↓SOD, ↓CAT, ↓GPx and glutathione reductase of all types of samples in CP; vitamin C and vitamin E of all types of samples in CP (except for reduced glutathione in the gingival tissues).	Panjamurthy et al., 2005
SOD; GPx; albumins; uric acid	Saliva	42 CP; 21 controls	↑SOD (*p* = 0.021) and ↑GPx (*p* = 0.000) in CP; ↓albumins in CP (*p* = 0.039); GPx, albumins and uric acid increases (*p* < 0.001), and SOD decreases (*p* < 0.005) after therapy.	Novakovic et al., 2014
GPx; uric acid	Saliva	20 CP; 20 controls	↓GPx and ↓uric acid in CP (*p* < 0.05); uric acid negatively correlates with C-terminal telopeptide of type I collagen and matrix metalloproteinases-8 (*p* < 0.05).	Miricescu et al., 2014
SOD; CAT; glutathione; total thiol	Gingival tissue; blood	35 CP (20 smokers, 10 non-smokers)	↑CAT and ↑total thiol of all types of samples in smokers; ↓glutathione of gingival tissue in smokers; ↓SOD of all types of samples in smokers.	Garg et al., 2006
Urate; vitamin A; vitamin C; vitamin E; thiols; bilirubin; cholesterol; thiglycerides; albumin	Serum	256 participants	Vitamin A (*p* < 0.0001), urate (*p* < 0.0001) and thiols (*p* < 0.01) are influenced by gender.	Maxwell et al., 2006
SOD	Gingival tissue	34 CP (17 with type 2 diabetics, 17 systemically healthy); 35 controls (18 with type 2 diabetics, 17 systemically healthy)	↓SOD in CP than controls (*p* < 0.05); ↑SOD in participants with diabetics than systemically healthy participants.	Akalin et al., 2008
SOD	GCF; saliva	60 smokers; 10 non-smokers	↓SOD of all types of samples in smokers than controls, and correlates with the extent of smoking.	Agnihotri et al., 2009
SOD	GCF; saliva	60 CP (33 pregnant, 27 non-pregnant); 18 gingivitis (pregnant); 46 controls (21 pregnant, 25 non-pregnant);	↑clinical parameters and ↓SOD in pregnancy, especially for the last phase of pregnancy	Akalin et al., 2009
SOD; CAT; GPx	Serum; gingival tissue	49 CP (23 smokers, 23 former smokers, 20 non-smokers); 20 controls (non-smokers)	↓SOD, ↓CAT, and ↓GPx of gingival tissue in CP than controls (*p* < 0.01); ↓SOD, ↓CAT, and ↓GPx of gingival tissue in CP (non-smokers) than CP (smokers and former smokers) (*p* < 0.01).	Tonguc et al., 2011

*SOD, superoxide dismutase; CAT, catalase; GPx, glutathione peroxidase; GCF, gingival crevicular fluid; CP, chronic periodontitis*.

SOD and CAT activities were measured in human gingival tissue and these activities were found to be reduced with the increasing periodontal pocket depth (Ellis et al., 1998). The activities of SOD and GPx in saliva were decreased in periodontitis patients (Canakci et al., 2009). Additionally, this study suggested a significant negative relationship between the level of 8-OHdG and MDA and the activities of SOD and GPx, whereas no correlation between clinical parameters and the enzymatic antioxidants activities was observed. Another study showed that the activities of antioxidant enzymes SOD, CAT, and glutathione reductase in saliva of periodontitis patients exhibited a significant negative correlation with periodontal parameters (Trivedi et al., 2015). In contrast to these studies, Panjamurthy et al. showed that activities of enzymatic antioxidants including SOD, CAT measured in plasma, erythrocytes and gingival tissues were elevated in periodontitis, whereas activities of non-enzymatic antioxidants including vitamins E, vitamin C, and reduced glutathione were decreased in periodontitis (Panjamurthy et al., 2005). Similarly, another study by Novakovic et al. showed higher activities of enzymatic antioxidants including SOD and GPx and lower activities of non-enzymatic antioxidants in saliva of periodontitis patients (Novakovic et al., 2014). Furthermore, periodontal treatment resulted in elevating the activities of albumins, uric acid, and GPx and decreasing the activity of SOD (Novakovic et al., 2014). The activity of uric acid was found to be lower in saliva of periodontitis patients and it was shown to be negatively correlated with bone resorption biomarkers such as C-terminal telopeptide of type I collagen and matrix metalloproteinases-8 (Miricescu et al., 2014). Therefore, current results on the relationship between periodontal status and enzymatic antioxidants activity are contradictory. Meta-analysis performed with 6 articles investigating the levels of circulating SOD found no significant difference in this parameter between periodontitis patients and healthy controls (Liu et al., 2014). In contrast, the results for non-enzymatic antioxidants are rather consistent and they suggest that the decreased activities of non-enzymatic antioxidants are associated with periodontitis. Thus, more additional well-designed studies are still required to clarify the relationship between enzymatic antioxidant activities and periodontitis.

Similarly to ROS production, numerous studies indicate that the changes in the activity of antioxidants in periodontitis are influenced by systemic conditions (Garg et al., 2006; Maxwell et al., 2006; Akalin et al., 2008, 2009; Agnihotri et al., 2009; Tonguc et al., 2011; Duarte et al., 2012; Trivedi et al., 2014). One study showed that women have lower activity of vitamin A and urate in serum than men (Maxwell et al., 2006). Gingival activities of specific antioxidants like SOD, CAT, and GPx could be increased by smoking among people with periodontitis, and these changes were considered as a protective or adoptive mechanism (Tonguc et al., 2011). In contrast, another study indicated a compromised activity of gingival SOD and glutathione in smokers (Garg et al., 2006). Smoking is also associated with decreased levels of SOD in GCF and saliva in both periodontitis patients and healthy individuals (Agnihotri et al., 2009). Diabetes mellitus can increase the activity of SOD and gene expression of SOD1 in gingival tissue of periodontitis patients (Akalin et al., 2008; Duarte et al., 2012). However, higher activities of SOD, CAT and glutathione reductase were found in saliva and plasma of systemically and periodontally healthy individuals compared to those with CP and/or diabetes mellitus (Trivedi et al., 2014). Activities of SOD were also found to be decreased by pregnancy among periodontitis patients (Akalin et al., 2009).

Antioxidants present a strong defense function against ROS; therefore, numerous studies tried to examine the application of antioxidants in the treatment of periodontitis. It has been shown that supplemental periodontal treatments with antioxidants like vitamin E, taurine and lycopene result in improved clinical periodontal parameters, higher activities of local and systemic antioxidants, and lower levels of local and systemic ROS compared with conventional periodontal treatment (Arora et al., 2013; Singh et al., 2014; Sree and Sethupathy, 2014). A recent review concluded a useful effect of vitamin C on maintaining periodontal health for elderly people (Alagl and Bhat, 2015). Another recent review focused on the effects of the complimentary use of lycopene, vitamin C, vitamin E, capsules with fruits/vegetables/berry and dietary interventions to periodontal therapy (Muniz et al., 2015). It confirmed that only the use of lycopene and vitamin E is associated with improved clinical parameters (Muniz et al., 2015). These results indicate a promising use of antioxidants for periodontitis treatment, which could be beneficial for both periodontal status and systemic oxidative status.

## Total antioxidant capacity, total oxidant status and oxidative stress index

### Total antioxidant capacity

The antioxidant system is highly complex and therefore the measurement of total antioxidant capacity (TAOC) was developed as a cost-effective instrument to assess the activity of the whole antioxidant system (Chapple et al., 1997). Most of the related studies suggested that periodontitis is associated with compromised local TAOC (Brock et al., 2004; Chapple et al., 2007a; Guentsch et al., 2008; Baltacioglu et al., 2014b; Baser et al., 2015; Zhang et al., 2016; Ahmadi-Motamayel et al., 2017). Moreover, some studies also indicated that periodontitis could influence the circulating TAOC (Brock et al., 2004; Chapple et al., 2007b; Abou Sulaiman and Shehadeh, 2010; D'Aiuto et al., 2010; Baltacioglu et al., 2014b; Thomas et al., 2014; Baser et al., 2015; Ahmadi-Motamayel et al., 2017). TAOC in plasma and saliva was shown to correlate with periodontal parameters (Baser et al., 2015; Zhang et al., 2016). However, there are contradictory data on the question whether periodontal treatment can improve local and/or circulating compromised TAOC (Guentsch et al., 2008; D'Aiuto et al., 2010; Novakovic et al., 2014; Thomas et al., 2014). Therefore, additional controlled studies on the effect of periodontal therapy on local and systemic TAOC are required. A recent study showed no relationship between TAOC and bacterial load in periodontitis suggesting that the changes of TAOC could be related to the host immune response rather than to the bacterial load (Zhang et al., 2016).

TAOC associated with periodontitis can be affected by systemic conditions like gender, smoking, pregnancy, and systemic diseases (Brock et al., 2004; Buduneli et al., 2006; Maxwell et al., 2006; Chapple et al., 2007a; Akalin et al., 2009; Pendyala et al., 2013a,b; Bakhtiari et al., 2015; Ahmadi-Motamayel et al., 2017). Some studies suggested that men have higher serum TAOC than women (Brock et al., 2004; Maxwell et al., 2006; Chapple et al., 2007a). One study also showed similar difference in saliva TAOC (Maxwell et al., 2006). One study showed that lower TAOC in serum and GCF was also associated with pregnancy, especially in the last trimester, and within the pregnant women decreasing TAOC was correlated to deteriorating periodontal status (Akalin et al., 2009). There is one study indicating that salivary TAOC among smokers is significantly lower than that among non-smokers; however, this study did not consider the worse periodontal status of smokers (Bakhtiari et al., 2015). Another study found that neither the gingivitis nor smoking status have influence on salivary TAOC (Buduneli et al., 2006). Further studies showed that both periodontitis and diabetes mellitus could contribute to lower TAOC in saliva, and decreased TAOC in saliva was also associated with periodontal status among people with diabetes mellitus (Pendyala et al., 2013a,b).

### TOS and OSI

In 2005, an assay based on the oxidation of ferrous ion to ferric ion in the presence of various oxidant species in acidic medium was introduced to measure the total oxidative status (Erel, 2005). Differently to previous methods focused on specific ROS or ROS products, this method could be used as a stable, cost-efficient and convenient measurement of the whole oxidant status. Another parameter called oxidative stress index (OSI), which is calculated as TOS/TAOC, was also introduced to show the level of oxidative stress with the balance of antioxidants (Erel, 2005).

These two parameters have been widely used to measure whole oxidative stress associated with periodontitis. Studies have shown that periodontitis is associated with higher value of TOS and OSI in GCF, saliva and serum (Erel, 2005; Akalin et al., 2007; Baltacioglu et al., 2014a,b), and these levels can also be reduced by periodontal treatment (Wei et al., 2010; Akpinar et al., 2013). Aggressive periodontitis was shown to be associated with significantly higher values of TOS and OSI compared to chronic periodontitis (Baltacioglu et al., 2014a,b). One study had even proposed OSI as a new biomarker for periodontitis based on its significant correlation with clinical parameters of periodontitis (Baltacioglu et al., 2014b). However, in contrast to this observation, our previous study did not show any significant difference in salivary TOS between generalized severe periodontitis patients and healthy controls (Zhang et al., 2016). This discrepancy might be due to the less restricted selection of periodontitis patients and indicates the limited utilization of salivary TOS as a marker of periodontitis.

TOS and OSI were also used to show the interaction between periodontitis and systemic conditions. One study showed that rheumatoid arthritis had no significant impact on local and systemic OSI of people with periodontitis (Esen et al., 2012). In contrast, another study showed that although rheumatoid arthritis or periodontitis have limited effect on serum OSI, individuals with both rheumatoid arthritis and periodontitis showed significant higher serum OSI compared to systemic and periodontally healthy individuals (Sezer et al., 2013). Another systemic disease familial Mediterranean fever was also shown to affect the local OSI of periodontitis patients (Bostanci et al., 2014). One study suggested that people with obesity were more likely to have higher value of TOS and OSI in serum and GCF and were predisposed to periodontitis (Dursun et al., 2016). TOS or OSI can also be used as the measurements of the effectiveness of newly developed periodontal therapy. Particularly, studies on rats show that boric acid, sumac extract and low-dose doxycycline could significantly reduce the oxidative stress indicated by OSI or TOS (Balci Yuce et al., 2014; Yagan et al., 2014; Saglam et al., 2015; Kose et al., 2016).

Summarizing, TOS and OSI can show the association between increased local and systemic oxidative stress and deteriorated periodontal status; however, their sensitivity needs to be further tested. Furthermore, these two measurements have the potential to evaluate the interaction between periodontal and systemic status and the effectiveness of periodontal treatment.

## Conclusions

It has been confirmed that periodontitis is associated with a hyperactivity of peripheral blood neutrophils, which are supposed to be the predominant source of ROS. Recent reports suggest that hyperactivity of neutrophils is likely to be a host-immune reaction to the inflammation of periodontitis, which might be also genetically predisposed. Numerous studies suggested that periodontitis could contribute to both local and systemic oxidative stress. Products of lipid peroxidation, protein damage and DNA damage can be used as the biomarkers of oxidative stress associated with periodontitis. Local and systemic activities of antioxidants can also be influenced by periodontitis. Some studies suggested decreased activities of enzymatic antioxidants, like SOD and CAT, are associated with periodontitis, whereas others showed increased activities of enzymatic antioxidants among people with periodontitis as a protective reaction. The results for non-enzymatic antioxidants as well as TAOC are consistent and indicate compromised antioxidant capacity in periodontitis patients. Different antioxidants have been applied as supplements to the conventional periodontal treatment and optimistic results were obtained, which provides new possibilities in the periodontal therapy. In recent years, TOS and OSI have been used more and more to evaluate total oxidative status or oxidative stress associated with periodontitis. Studies measuring these parameters also confirmed increased local and systemic oxidative stress was associated with the inflammation resulted from periodontitis, but their sensitivity to be used as biomarkers for oxidative stress associated with periodontitis needs to be further verified.

Our review focused on the presence of oxidative stress associated with periodontitis, especially on the relationship between the local and systemic biomarkers of oxidative stress and periodontitis, giving us an implication of pathogenesis of periodontitis through oxidative stress, close relationship between periodontitis and systemic conditions, and promising therapeutic strategies involving antioxidants.

## Author contributions

Conception and design: XR-F, OA, and YW. Search references: YW. Drafted manuscript: YW. Critically revised the manuscript: YW, OA, and XR-F.

### Conflict of interest statement

The authors declare that the research was conducted in the absence of any commercial or financial relationships that could be construed as a potential conflict of interest.
